# Accuracy of Vitalograph lung monitor as a screening test for COPD in primary care

**DOI:** 10.1038/s41533-019-0158-2

**Published:** 2020-01-03

**Authors:** A. P. Dickens, D. A. Fitzmaurice, P. Adab, A. Sitch, R. D. Riley, A. Enocson, R. E. Jordan

**Affiliations:** 1 Institute of Applied Health Research, University of Birmingham, Birmingham, UK; 2 Warwick Medical School – Health Sciences, University of Warwick, Coventry, UK; 3 Centre for Prognosis Research, Research Institute for Primary Care and Health Sciences, Keele University, Keele, UK

**Keywords:** Diagnosis, Chronic obstructive pulmonary disease

## Abstract

Microspirometry may be useful as the second stage of a screening pathway among patients reporting respiratory symptoms. We assessed sensitivity and specificity of the Vitalograph® lung monitor compared with post-bronchodilator confirmatory spirometry (ndd Easy on-PC) among primary care chronic obstructive pulmonary disease (COPD) patients within the Birmingham COPD cohort. We report a case–control analysis within 71 general practices in the UK. Eligible patients were aged ≥40 years who were either on a clinical COPD register or reported chronic respiratory symptoms on a questionnaire. Participants performed pre- and post-bronchodilator microspirometry, prior to confirmatory spirometry. Out of the 544 participants, COPD was confirmed in 337 according to post-bronchodilator confirmatory spirometry. Pre-bronchodilator, using the LLN as a cut-point, the lung monitor had a sensitivity of 50.5% (95% CI 45.0%, 55.9%) and a specificity of 99.0% (95% CI 96.6%, 99.9%) in our sample. Using a fixed ratio of FEV_1_/FEV_6_ < 0.7 to define obstruction in the lung monitor, sensitivity increased (58.8%; 95% CI 53.0, 63.8) while specificity was virtually identical (98.6%; 95% CI 95.8, 99.7). Within our sample, the optimal cut-point for the lung monitor was FEV_1_/FEV_6_ < 0.78, with sensitivity of 82.8% (95% CI 78.3%, 86.7%) and specificity of 85.0% (95% CI 79.4%, 89.6%). Test performance of the lung monitor was unaffected by bronchodilation. The lung monitor could be used in primary care without a bronchodilator using a simple ratio of FEV_1_/FEV_6_ as part of a screening pathway for COPD among patients reporting respiratory symptoms.

## Introduction

Chronic obstructive pulmonary disease (COPD) is one of the most common long-term respiratory conditions with rising burden and mortality worldwide.^1–3^ It is characterised by increasing breathlessness and decline in lung function, punctuated by episodes of acute exacerbations that often lead to hospital admission and result in poor prognosis and gradual deterioration of quality of life.^4^ Annual healthcare and societal costs of COPD in Europe are estimated to be €48.4 billion.^5^ Despite the high burden of disease, the large majority of patients with COPD remain undiagnosed^6^ while experiencing significant morbidity,^7^ resulting in calls to improve early diagnosis.^8,9^ Early diagnosis could focus smoking cessation support and allow prescription of treatments that have been shown to reduce risk of exacerbation in those with COPD, thus has the potential to slow disease progression.

Screening programmes are not yet recommended, partly because of lack of evidence of the long-term benefits,^10,11^ a view which is upheld in the most recent UK National Screening Committee report.^12^ However, there are also uncertainties around the performance of available screening tests, including symptom or risk assessment questionnaires and lung function-based measures, alone or in combination.^12,13^ A recent study compared different screening strategies among current smokers, against post-bronchodilator spirometry. This concluded that microspirometry or peak flow meters had the best performance, but interpretation was limited by a small sample size and low-quality spirometry data.^14^ Microspirometers are small relatively inexpensive handheld devices that measure forced expiratory volume in 1 s (FEV_1_) and in 6 s (FEV_6_). While this is not a substitute for confirmatory spirometry, which is more time consuming and measures FEV_1_ and forced vital capacity (FVC), usually after bronchodilation, the FEV_1_/FEV_6_ ratio could be used as a pragmatic initial screening test to identify patients requiring confirmatory spirometry. Microspirometry can be undertaken in office settings and requires less time and patient effort.^15–18^

Over the past decade, several studies have explored the accuracy of microspirometers in detecting airflow obstruction.^14,19–27^ However, none of the studies considered the use of microspirometers as the second stage of a screening pathway. Microspirometry as a screening tool is usually performed without bronchodilation, as this contributes to time savings and avoids the need for Salbutamol. However, it remains uncertain how microspirometry performance differs when conducted pre- and post-bronchodilator. Finally, there is little consensus regarding the optimal FEV_1_/FEV_6_ cut-point for referral to confirmatory spirometry, with recent studies suggesting ratios of <0.73,^22^ <0.75^21^ and <0.78.^19^

To address the current evidence gaps, we conducted a study in primary care patients with existing respiratory symptoms, including those pre-screened in our linked trial. We aimed to assess the test performance of a microspirometer (Vitalograph Lung monitor) against confirmatory post-bronchodilator spirometry (ndd Easy on-PC) and explore the effect of using pre- or post-bronchodilator microspirometer data, the impact of using different airflow obstruction criteria and optimal cut-points.

## Results

**Fig. 1:**
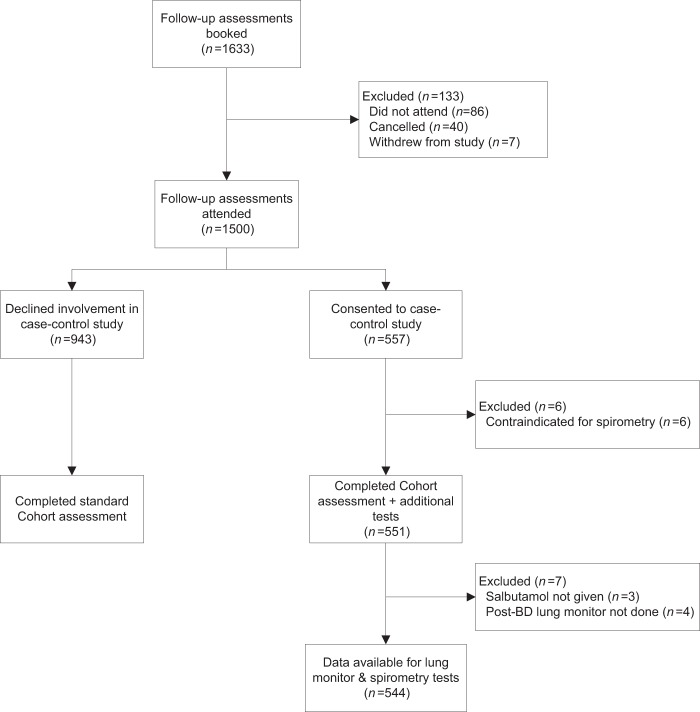
Flow of the participants.

Follow-up assessments were booked for 1633 participants. Out of the 1500 participants who attended the assessment, 551 took part in the case–control study. Lung monitor and spirometry test data were available for a total of 544 participants (Fig. 1).

Of those, 349 (64.2%) were male, the mean age was 69.6 (9.1) years, 517 (96.3%) were White British, 382 (74.3%) were overweight/obese and 472 (88.1%) had a positive smoking history. Similar proportions of participants reported Medical Research Council (MRC) Dyspnoea scores of 1–2 and 3–5, one fifth (19.7%) had CAT scores representing high impact on daily life and over half the sample (57.3%) were retired (Table 1).

**Table 1:** Description of analysis sample, stratified by cases and controls.

	Analysis sample (*n* = 544)	Cases; <LLN (*n* = 337)	Controls; ≥LLN (*n* = 207)
Demographic and lifestyle information			
Sex; *n* (%) male	349/544 (64.2)	230/337 (68.3)	119/207 (57.5)
Age, mean (SD)	69.6 (9.1)	70.4 (7.9)	68.2 (10.6)
Ethnicity; *n* (%)			
White British	517/537 (96.3)	322/332 (97.0)	195/205 (95.1)
Other	20/537 (3.7)	10/332 (3.0)	10/205 (4.9)
Weight; *n* (%)			
Underweight (BMI <18.5)	9/514 (1.8)	7/318 (2.2)	2/196 (1.0)
Healthy (≥18.5 and <25)	123/514 (23.9)	85/318 (26.7)	38/196 (19.4)
Overweight (≥25 and <30)	194/514 (37.7)	118/318 (37.1)	76/196 (38.8)
Obese (≥30)	188/514 (36.6)	108/318 (34.0)	80/196 (40.8)
BMI, mean (SD) *n* = 514	28.6 (5.4)	28.1 (5.4)	29.4 (5.4)
Smoking status; *n* (%)			
Current smoker	111/536 (20.7)	79/332 (23.8)	32/204 (15.7)
Ex-smoker	361/536 (67.4)	221/332 (66.6)	140/204 (68.6)
Never smoker	64/536 (11.9)	32/332 (9.6)	32/204 (15.7)
Employment status; *n* (%)			
Employed	88/534 (16.5)	45/334 (13.5)	43/200 (21.5)
Unemployed	140/534 (26.2)	94/334 (28.4)	46/200 (23.0)
Retired	306/534 (57.3)	195/334 (58.4)	111/200 (55.5)
Airflow obstruction			
GOLD stage if <LLN			
1 (FEV_1_ ≥80% pred)	87/337 (25.8)	87/337 (25.8)	n/a
2 (50–79%)	177/337 (52.5)	177/337 (52.5)	n/a
3 (30–49%)	59/325 (18.1)	61/337 (18.1)	n/a
4 (<30%)	12/325 (3.6)	12/337 (3.6)	n/a
Self-reported health and healthcare usage			
MRC; *n* (%)			
1–2	219/427 (51.3)	116/255 (45.5)	103/172 (59.9)
3–5	208/427 (48.7)	139/255 (54.5)	69/172 (40.1)
CAT impact level; *n* (%)			
Low (0–9)	180/406 (44.3)	100/245 (40.8)	80/161 (49.7)
Medium (10–20)	146/406 (36.0)	93/245 (38.0)	53/161 (32.9)
High (21–30)	69/406 (17.0)	47/245 (19.2)	22/161 (13.7)
Very high (31–40)	11/406 (2.7)	5/245 (2.0)	6/161 (3.7)
Asthma; *n* (%) yes	198/538 (36.8)	129/334 (38.6)	69/204 (33.8)
CVD^a^; *n* (%) yes	159/538 (29.6)	94/334 (28.1)	65/204 (31.9)
Other comorbidities^b^; *n* (%) ≥1	302/540 (55.9)	184/336 (54.8)	111/204 (57.8)
Exacerbations in last 12 m; *n* (%) yes	247/543 (45.5)	182/336 (54.2)	65/207 (31.4)
Respiratory hospitalisation in last 2 years; *n* (%) yes	37/544 (6.8)	27/337 (8.0)	10/207 (4.8)

LLN = below the 5th percentile of the predicted FEV_1_/FVC ratio using the NHANES III equations

^a^CVD was defined as self-reported coronary heart disease, heart failure or other heart condition

^b^Other comorbidities were defined as self-reported clinician diagnosis of diabetes, osteoporosis, depression, fracture

A total of 337 (62.0%) participants had airflow obstruction according to the reference test, with over three quarters (*n* = 264, 78.3%) representing GOLD stage I and II i.e. mild/moderate COPD (Table 1). In comparison with controls, the cases were slightly older (70.4 vs 68.2 years), more likely to be male (68.3% vs 57.5%), a higher proportion had a positive smoking history (90.4% vs 84.3%) and MRC Dyspnoea scores of 3–5 (54.5% vs 40.1%) (Table 1).

Nearly half of the participants (45.5%) reported exacerbations in the past 12 months and 37 (6.8%) reported a respiratory hospitalisation in the past 2 years (Table 1). Cases reported approximately twice as many exacerbations as controls (54.2% vs 31.4%) in the past 12 months.

### Screening accuracy of the pre-bronchodilator lung monitor

Using lower limit of normal (LLN; i.e. the lower 5th percentile) as the cut-off for a positive result, the pre-bronchodilator lung monitor had sensitivity of 50.5% (95% confidence interval (CI) 45.0, 55.9) and specificity of 99.0% (95% CI 96.6, 99.9) (Table 2). The positive predictive value of the lung monitor was estimated to be 76.9% (95% CI 55.7, 89.8) for a population prevalence of 6%, dropping to 61.8% (95% CI 37.8, 81.1) for a population prevalence of 3% and rising to 85.3% (95% CI 68.6, 93.9) for a population prevalence of 10%.

**Table 2:** Pre-BD lung monitor (FEV_1_/FEV_6_ < LLN) against post-BD confirmatory spirometry (FEV_1_/FVC < LLN).

	Spirometry +ve	Spirometry −ve	
Lung monitor +ve	170 (50.5%)	2 (1.0%)	172
Lung monitor −ve	167 (49.6%)	205 (99.0%)	372
	337	207	544

FEV_1_ measurements from both devices were highly correlated (*r* = 0.97; *p* < 0.001), with the Bland–Altman plot demonstrating good agreement (Supplementary Fig. 1). Comparison of FEV_6_ from both devices again revealed high correlation (*r* = 0.95; *p* < 0.001), though agreement was lower, indicating that the lung monitor systematically underestimated FEV_6_ values by −0.37 litres (Supplementary Fig. 2).

The lung monitor had high discriminatory accuracy (*C* = 0.90; 95% CI 0.88, 0.93) between cases and controls according to confirmatory spirometry FEV_1_/FVC < LLN (Supplementary Fig. 3).

### Screening accuracy of the post-bronchodilator lung monitor

Using post-bronchodilator data for the lung monitor, the sensitivity was 46.6% (95% CI 41.2%, 52.1%) and specificity was 97.1% (95% CI 93.8%, 98.9%) for detecting airflow obstruction (Table 3). The positive predictive value of the lung monitor was estimated to be 50.6% (95% CI 42.6, 58.6) for a population prevalence of 6%.

**Table 3:** Post-BD lung monitor (FEV_1_/FEV_6_ < LLN) against post-BD confirmatory spirometry (FEV_1_/FVC < LLN).

	Spirometry +ve	Spirometry −ve	
Lung monitor +ve	157 (46.6%)	6 (2.9%)	163
Lung monitor −ve	180 (53.4%)	201 (97.1%)	381
	337	207	544

Comparison of pre- and post-bronchodilator lung monitor test accuracy revealed a borderline significant difference favouring pre-bronchodilator of 3.9% in sensitivity (7.8%, −0.05%); *p* = 0.04, but no statistically significant evidence of a difference in specificity (1.9% (−0.9%, 4.7%)); *p* = 0.10.

Lung monitor and confirmatory spirometry tests were also highly correlated for post-bronchodilator FEV_1_ (*r* = 0.97; *p* < 0.001), with the Bland–Altman plot again demonstrating good agreement (Supplementary Fig. 4). Comparison of post-bronchodilator FEV_6_ again revealed high correlation (*r* = 0.97; *p* < 0.001), though agreement was lower, indicating that the lung monitor systematically underestimated FEV_6_ values by −0.28 litres (Supplementary Fig. 5).

Discriminatory accuracy of the post-bronchodilator lung monitor was identical to that based on pre-bronchodilator data (*C* = 0.90; 95% CI 0.87, 0.92; Supplementary Fig. 6).

### Sensitivity analyses: optimal cut-points for lung monitor FEV_1_/FEV_6_ ratio, relative to confirmatory spirometry FEV_1_/FVC < LLN

In light of comparable test accuracy of the lung monitor based on pre- and post-bronchodilator data, we explored optimal FEV_1_/FEV_6_ cut-points using pre-bronchodilator tests (Table 4).

**Table 4:** Screening accuracy of pre-bronchodilator lung monitor FEV_1_/FEV_6_ cut-points, against post-BD confirmatory spirometry (FEV_1_/FVC < LLN).

	TP	FP	TN	FN	Sens (95% CI)	Spec (95% CI)	PPV (3% prevalence)	PPV (6% prevalence)	PPV (10% prevalence)	Proportion referred to diagnostic test^a^	Proportion of true cases missed^a^
<0.4	6	0	207	331	1.79 (0.7, 3.8)	100 (98.2, 100)	—	—	—	1.1%	98.2%
<0.5	34	0	207	303	10.1 (7.1, 13.8)	100 (98.2, 100)	—	—	—	6.3%	89.9%
<0.6	88	1	206	249	26.1 (21.5, 31.1)	99.5 (97.3, 100)	0.63 (0.19, 0.92)	0.78 (0.33, 0.96)	0.86 (0.46, 0.98)	16.4%	73.9%
<0.7	197	3	204	140	58.5 (53.0, 63.8)	98.6 (95.8, 99.7)	0.56 (0.39, 0.70)	0.72 (0.57, 0.83)	0.82 (0.70, 0.90)	36.8%	41.5%
<0.71	207	4	203	130	61.4 (56.0, 66.6)	98.1 (95.1, 99.5)	0.50 (0.38, 0.61)	0.67 (0.56, 0.77)	0.78 (0.69, 0.85)	38.8%	38.6%
<0.72	220	7	200	117	65.3 (59.9, 70.4)	96.6 (93.2, 98.6)	0.37 (0.31, 0.44)	0.55 (0.48, 0.62)	0.68 (0.62, 0.74)	41.7%	34.7%
<0.73	234	9	198	103	69.4 (64.2, 74.3)	95.7 (91.9, 98.0)	0.33 (0.29, 0.38)	0.50 (0.45, 0.56)	0.64 (0.59, 0.69)	44.7%	30.6%
<0.74	244	12	195	93	72.4 (67.3, 77.1)	94.2 (90.1, 97.0)	0.28 (0.25, 0.31)	0.44 (0.41, 0.48)	0.58 (0.54, 0.62)	47.1%	27.6%
<0.75	253	17	190	84	75.1 (70.1, 79.6)	91.8 (87.2, 95.1)	0.22 (0.20, 0.24)	0.37 (0.34, 0.39)	0.50 (0.48, 0.53)	49.6%	24.9%
<0.76	264	25	182	73	78.3 (73.6, 82.6)	87.9 (82.7, 92.0)	0.17 (0.16, 0.18)	0.29 (0.28, 0.31)	0.42 (0.40, 0.44)	53.1%	21.7%
<0.77	273	29	178	64	81.0 (76.4, 85.1)	86.0 (80.5, 90.4)	0.15 (0.14, 0.16)	0.27 (0.26, 0.28)	0.39 (0.38, 0.41)	55.5%	19.0%
<0.78	279	31	176	58	82.8 (78.3, 86.7)	85.0 (79.4, 89.6)	0.15 (0.14, 0.15)	0.26 (0.25, 0.27)	0.38 (0.37, 0.39)	57.0%	17.2%
<0.79	287	41	166	50	85.2 (80.9, 88.8)	80.2 (74.1, 85.4)	0.12 (0.11, 0.12)	0.22 (0.21, 0.22)	0.32 (0.31, 0.33)	60.3%	14.8%
<0.8	295	48	159	42	87.5 (83.5, 90.9)	76.8 (70.5, 82.4)	0.10 (0.10, 0.11)	0.19 (0.19, 0.20)	0.30 (0.29, 0.30)	63.1%	12.5%
<0.9	333	182	25	4	98.8 (97.0, 99.7)	12.1 (8.0, 17.3)	0.03 (0.03, 0.03)	0.07 (0.07, 0.07)	0.11 (0.11, 0.11)	94.7%	1.2%

*TP* true positives, *FP* false positives, *TN* true negatives, *FN* false negatives, *Sens* sensitivity, *Spec* specificity, *PPV* positive predictive value

^a^Assuming 6% COPD prevalence

Using an FEV_1_/FEV_6_ cut-point of <0.7 to define a positive test for the lung monitor, sensitivity increased to 58.5% (95% CI 53.0, 63.8), specificity was 98.6% (95% CI 95.8, 99.7) and the positive predictive value increased to 72.0% (95% CI 57.4, 83.1) for a population prevalence of 6% (Table 4). However, using a fixed ratio had little effect on the discriminatory accuracy of the lung monitor (*C* = 0.91; 95% CI 0.89, 0.94; Supplementary Fig. 7).

In our sample, an FEV_1_/FEV_6_ cut-point of <0.78 had the best overall test performance with sensitivity of 82.8% (95% CI 78.3%, 86.7) and specificity of 85.0% (95% CI 79.4%, 89.6%). Using this cut-point would result in the lung monitor only missing 17.2% of true positives and correctly identifying the majority of patients without the disease. Furthermore, this cut-point would result in 57% of those screened requiring confirmatory spirometry. The positive predictive value for a population COPD prevalence of 6% was estimated to be 26.1% (95% CI 25.0, 27.2) meaning that around one in four patients referred for confirmatory spirometry would result in a diagnosis.

The above pattern was broadly similar when analyses were repeated using the fixed ratio to define obstruction for confirmatory spirometry (FEV_1_/FVC < 0.7), though sensitivity was slightly lower at each cut-point and specificity remained at 100% until FEV_1_/FEV_6_ > 0.7 (Supplementary Table 1). These analyses may reflect the test performance when using the simpler criterion for the lung monitor in countries defining airflow obstruction as FEV_1_/FVC < 0.7, such as the UK.^28^

## Discussion

We found that the lung monitor has high discriminatory accuracy among patients with existing chronic respiratory symptoms. This supports its suitability, either alone or perhaps in combination with a symptom questionnaire, as a screening test prior to confirmatory spirometry. We further demonstrated that using a bronchodilator with the lung monitor as part of screening offers no performance advantage.

Importantly, the lung monitor demonstrated good test performance despite being delivered with minimal coaching and only requiring a maximum of three blows, rather than the possible six blows to achieve repeatability with confirmatory spirometry.

Using pre-bronchodilator FEV_1_/FEV_6_ < LLN, the lung monitor missed half of COPD cases identified by FEV_1_/FVC < LLN from confirmatory spirometry but detected virtually all non-COPD cases correctly. When using pre-bronchodilator FEV_1_/FEV_6_ < 0.70, the lung monitor detected a higher proportion of true positives, the same proportion of true negatives and the discriminatory accuracy remained constant (*C* = 0.90 vs *C* = 0.91). Given the added complexity of applying LLN to the lung monitor as it is not connected to computer software, it appears justifiable to apply an FEV_1_/FEV_6_ fixed ratio to the lung monitor for purposes of screening, while maintaining the LLN for diagnosing and monitoring COPD.^29–32^

Test performance varied considerably depending on the specified cut-point of the pre-bronchodilator FEV_1_/FEV_6_ ratio. Our proposed optimal cut-point of <0.78 was similar to previous studies, which had suggested using cut-points of <0.75,^21^ <0.78^19^ and <0.80.^20^ The sensitivity and specificity of the lung monitor in our sample was acceptable for a screening test, missing <20% of COPD cases, while 1 in 4 patients of the 57% referred for confirmatory spirometry were true positives and therefore would be eligible for diagnosis and relevant treatment. While FEV_1_/FEV_6_ < 0.78 appeared the most efficient in our sample, if the lung monitor were to be used as a screening test the cut-point could be modified according to the balance of acceptable false negative rates and availability of resources.

We have assessed the screening test performance of one type of microspirometer. One factor affecting accuracy may be the different lung function indices being measured: FEV_6_ by the lung monitor vs FVC by the ndd device (confirmatory spirometry). We assessed test performance of both devices using FEV_1_/FEV_6_ < LLN as the cut-off for a positive result, relative to confirmatory spirometry FEV_1_/FVC < LLN. The ndd device had sensitivity of 80.4% and specificity of 98.1%, compared with the lung monitor sensitivity of 50.5% and specificity of 99.0%. This suggests that the difference in indices only partly affects performance. Another important difference to consider is the type of sensor used in the two devices for flow/volume measurement (turbine in the lung monitor vs ultrasonic in the ndd), as evidence suggests a degree of inaccuracy in turbine devices.^33,34^

Our analysis sample had fewer controls than determined by our sample size calculation, containing 207 instead of 248. While the precision around specificity was reduced, the precision around sensitivity estimates was unaffected; the latter being arguably more important in the context of screening.

Using the LLN criteria to define cases in our primary analysis ensured an accurate assessment of lung function, without added ‘noise’ from misdiagnosed patients which can be introduced when using the FEV_1_/FVC < 0.7 ratio.^32^ As the majority of previous microspirometry test accuracy studies used the fixed ratio definition of obstruction,^19–21,24,25,27,35^ our study has made a valuable contribution to the evidence base.

Owing to the case–control study being nested within a larger COPD cohort study, the analysis sample had a higher prevalence of COPD and possibly more advanced disease than would be observed in an undiagnosed primary care population reporting respiratory symptoms. Therefore, our study is at potential risk of spectrum bias, as the reported sensitivities and specificities may not fully reflect the test performance of the lung monitor if used as a screening tool within symptomatic patients with lower prevalence of COPD. However, by using Bayes’ Theorem the reported post-test estimates were based on current UK COPD prevalence of 3–10%, mitigating against this risk.

Nearly a third of our sample was a screened population, suggesting that our findings will resonate with potential screening processes, as patients could be selected for microspirometry on the basis of symptom- or risk-based screening tests. Furthermore, the fact that we included patients with chronic respiratory symptoms and a range of lung function severities means that our results may apply to an undiagnosed population with a similar symptom profile. In addition, our study was not restricted to ever-smokers, unlike previous studies.^14,19,21,22,25^

For practical reasons, the same researcher administered both the lung monitor and confirmatory spirometry. Although researchers only recorded raw FEV_1_ and FEV_6_ lung monitor values and did not calculate obstruction from this first test, it is possible that researchers were not entirely blind when administering the confirmatory spirometry to the patient. While this introduced a risk of review bias, this was minimised as researchers received standardised training to give only brief instruction for lung monitor tests and proper coaching for confirmatory spirometry.

Most previous studies have either used only pre-bronchodilator microspirometry^19–21,23–26^ or post-bronchodilator microspirometry,^27^ and the only study to measure pre- and post-bronchodilator microspirometry did not report comparative test accuracy.^22^ By demonstrating the comparability of test performance irrespective of bronchodilation, our study supports the continued use of pre-bronchodilator microspirometers for screening purposes.

Participants performed three blows using the lung monitor, irrespective of blow quality as indicated by the device’s in-built quality alert, with the highest recorded readings being used for analyses. While this follows some previous studies,^23,25,26^ had we required all lung monitor blows to be technically valid^19,20,22^ we may have obtained greater FEV_1_ or FEV_6_ values for some participants. Furthermore, like most studies we did not assess within-participant repeatability across blows on the lung monitor, though this has been done in at least one study.^21^

The observed test performance of the lung monitor suggests that it could be reliably used as a screening tool in patients perceived to be at risk of COPD, to select those requiring confirmatory spirometry. The efficiency of the diagnostic spirometry test could therefore be substantially increased, by patients highly unlikely to have airflow obstruction being screened out in advance. Screening at-risk symptomatic patients with a lung monitor rather than referring all patients for confirmatory spirometry also represents financial savings, with the handheld device being approximately one tenth of the cost of diagnostic spirometers. Resource savings could be realised in practices irrespective of whether they conduct confirmatory spirometry ‘in house’ or refer patients to a lung function unit, as both models would reduce the number of patients performing this diagnostic test.

The ability to use a fixed ratio for the lung monitor rather than the LLN to assess airflow obstruction represents a time saving for clinicians, who would otherwise need to use software to refer to reference equations. The comparable test performance of the lung monitor irrespective of bronchodilation supports the use of pre-bronchodilator tests, further contributing to the efficiency and ease of the screening test, a key consideration in the context of time-pressured primary care consultations.

The lung monitor could potentially be administered by any member of a primary care team, as it is a simple device requiring minimal training. This would be beneficial in general practice where staff may be unfamiliar with the device^19^ and the simplicity may minimise the risk of becoming de-skilled in using the lung monitor, in contrast to confirmatory spirometry where clinicians’ skills can reduce over time if they do not perform the test regularly.^36^

The simplicity of the lung monitor, the minimal number of required blows and its good test performance suggests that it could be particularly useful as a screening test in patients with poor coordination or lower cognitive ability. Furthermore, our Patient Advisory Group preferred the lung monitor over other microspirometer models suggesting that it may be more acceptable to patients.

While we have suggested optimal cut-points based on the balance of sensitivity and specificity, in practice, the optimal cut-point would be determined by the clinical setting in which the lung monitor was being used. For example, in settings where access to quality confirmatory spirometry may not be available, particularly in low-resource settings, specificity of the lung monitor may be prioritised. In these settings, using thresholds with higher specificity could effectively exclude the majority of those with respiratory symptoms who do not have COPD, thus preventing overdiagnosis.

In addition to use as a screening tool, the accurate measurement of FEV_1_ may indicate that the device could be used to monitor obstruction severity or lung function decline among diagnosed COPD patients, for example during annual reviews. Further research would be needed to explore this, but the potential time and cost savings afforded by using the lung monitor instead of confirmatory spirometry may be attractive to General Practitioner practices, who would still obtain annual FEV_1_ values as recommended by bodies such as the National Institute for Health and Care Excellence in the UK.^28^

Future research could build on preliminary evidence regarding microspirometer screening strategies,^13,14^ which could be implemented in differing clinical or economic contexts. Using a combination of microspirometry and screening questionnaires for example may prove more efficient than microspirometry alone. Furthermore, rather than using one cut-point to identify patients requiring confirmatory spirometry, certain contexts may warrant using two cut-points to refer only those patients where there is uncertainty about their diagnosis. For example, in low- and middle-income countries where availability of confirmatory spirometry may be limited, a three-tiered approach may be plausible whereby the top proportion of patients are defined test negative, the bottom proportion are defined as test positive and the middle proportion are referred for confirmatory spirometry.

Our results show that the Vitalograph lung monitor, which is a cheap and simple device, has acceptable accuracy for use within a screening pathway for undiagnosed COPD among primary care patients with respiratory symptoms. We have established that the test performance of the lung monitor is unaffected by bronchodilation, and our optimum cut-point of FEV_1_/FVC < 0.78 supports previous studies, with no observed advantage of using LLN for this screening test. Our paper makes a valuable contribution to the evidence base concerning potential COPD screening tests, though more work is required to inform the need for a formal screening programme.

## Methods

### Study design

We conducted a prospective case–control study to evaluate the screening performance of the Vitalograph® lung monitor (Vitalograph Ltd, Buckingham, UK), nested within a large COPD Cohort study.

### Participant recruitment

Study participants were drawn from those attending for their 3-year follow-up assessment as part of the Birmingham COPD Cohort Study, which has been reported in detail elsewhere.^37^ In brief, participants were primary care patients aged ≥40 years, who either had previously clinically diagnosed COPD or had reported chronic respiratory symptoms as part of a case-finding trial.^38^ Participants from the case-finding trial were invited to join the Cohort study, irrespective of their spirometry results, if they reported chronic cough or phlegm for ≥3 months for at least 2 years, wheeze in the past 12 months or dyspnoea of MRC grade ≥2.

**Fig. 2:**
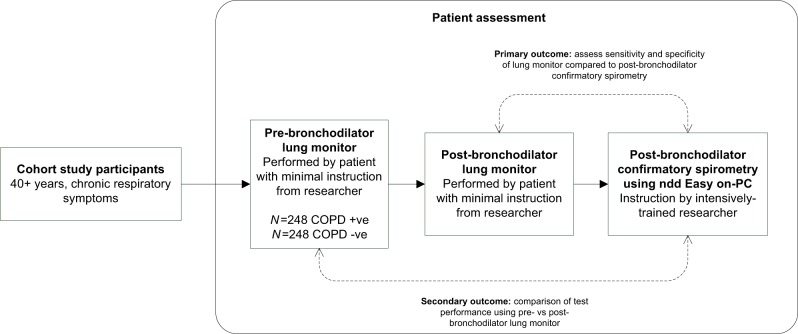
Case–control study design.

At the 3-year follow-up assessment visit, cohort participants were invited to take part in the additional tests for this case–control study (Fig. 2) and those who agreed were asked to sign a consent form. Those who declined to participate completed the standard Cohort assessment. The National Research Ethics Service Committee West Midlands, Solihull provided approval for both the Birmingham COPD Cohort (11/WM/0304) and the case-finding trial (11/WM/0403).

### Data collection and clinical measures

In addition to the lung function tests described below, participants underwent the standard Cohort follow-up assessment, which included various physiological and anthropometric measurements (height, weight, grip strength, exercise capacity) as well as completing questionnaires.

### Index test: lung monitor microspirometry

Participants received pre- and post-bronchodilator microspirometry with the Vitalograph lung monitor prior to confirmatory post-bronchodilator spirometry (Fig. 1). The lung monitor measured FEV_1_ and FEV_6_ in litres. In contrast to confirmatory spirometry, participants received minimal explanation or coaching when using the lung monitor. Researchers told participants to take a deep breath until lungs were full and blow into the mouthpiece as hard and fast as they could until being told to stop. Researchers demonstrated the correct technique once and then allowed the participant to perform the blows themselves, without additional coaching or encouragement. Participants performed three blows pre-bronchodilator and three blows post-bronchodilator. Technically unsatisfactory blows identified by the in-built quality assessment were recorded on the case report form, but participants were not asked to repeat the blow. The best FEV_1_ and FEV_6_ blows were used for analyses, irrespective of quality and which blow attempt they came from.

Positive test results were defined as being below the 5th percentile of the predicted pre-bronchodilator FEV_1_/FEV_6_ ratio (i.e. the LLN) using the NHANES III equations.^39^ Alternative positive test results were also pre-specified, including post-bronchodilator FEV_1_/FEV_6_ below the LLN, and various cut-points of the FEV_1_/FEV_6_ ratio.

### Reference test: post-bronchodilator confirmatory spirometry

Post-bronchodilator confirmatory spirometry was conducted according to American Thoracic Society and European Respiratory Society 2005 guidelines^40^ by trained researchers using the ndd Easy on-PC spirometer. Participants received 400 μg of Salbutamol and after waiting at least 20 min, performed a minimum of 3 (maximum of 6) blows until repeatability was achieved. Although the lung monitor and spirometry tests were administered by the same researcher, the tests were in effect administered blind of each other, as researchers did not record the FEV_1_/FEV_6_ ratio for the lung monitor before administering confirmatory spirometry.

Cases were defined as participants whose predicted FEV_1_/FVC ratio was below the LLN using the NHANES III equations, according to confirmatory spirometry. Participants not meeting this criterion formed the controls.

### Aims

The primary aim was to assess the pre-bronchodilator test accuracy (sensitivity and specificity) of the lung monitor (FEV_1_/FEV_6_) against post-bronchodilator confirmatory spirometry (FEV_1_/FVC), using the LLN definition of airflow obstruction.

We also aimed to assess the correlation and agreement between lung function measures from both devices and to compare test accuracy of pre- and post-bronchodilator lung monitor data. Finally, to identify the threshold that optimised sensitivity and specificity, we explored the effect of using different FEV_1_/FEV_6_ thresholds, including the fixed ratio of <0.7, to define a positive test result on the lung monitor.

### Sample size

We calculated that we required a sample size of 248 cases and 248 controls to detect an assumed sensitivity of 85%^21,27^ while ensuring the lower bound of the CI was >80%.

### Statistical analysis

We evaluated the diagnostic test accuracy of the lung monitor (index test) for all participants with complete data for the index and reference tests. We estimated sensitivity and specificity of the lung monitor using pre-bronchodilator data. We compared test accuracy of pre- and post-bronchodilator lung monitor blows, using McNemar’s test. Using continuous test values, we assessed the discriminatory accuracy of FEV_1_ and FEV_6_ measured by the lung monitor via receiver operating characteristic curve analysis. We then conducted sensitivity analyses using a fixed ratio definition of obstruction to identify a lung monitor FEV_1_/FEV_6_ optimal threshold.

To account for the case–control study design, post-test probabilities (herein referred to as positive predictive values (PPV)) were calculated using Bayes’ Theorem to reflect current COPD prevalence in the UK. For our tables and appendices, we calculated PPVs based on COPD prevalence among adults aged ≥40 years being 3–10%.^1,41^

All analyses were conducted in Stata SE v15.

The paper was written according to the STARD guidance^42^ for reporting studies of diagnostic accuracy.

## Supplementary information

Supplementary Information

## Data Availability

The data that support the findings of this study are available on request from the corresponding authors (A.P.D. or P.A.).

## Code availability

No custom code or mathematical algorithms were used in the analyses, which adopted only standard data manipulation and analysis within the statistical software.
