# Supplementary material for: Accuracy of Vitalograph lung monitor as a screening test for COPD in primary care
Source: NPJ Prim Care Respir Med. 2020 Jan 3;30:2. doi: 10.1038/s41533-019-0158-2 (PMC6941963; doi:10.1038/s41533-019-0158-2)
Supplement: Supplementary file 1 — Supplementary Information [file 41533_2019_158_MOESM1_ESM.pdf]

Supplementary table 1: Screening accuracy of pre-bronchodilator lung monitor FEV_1/FEV_6 cut-points, against post-BD confirmatory spirometry (FEV_1/FVC <0.7)

	TP	FP	TN	FN	Sens (95% CI)	Spec (95% CI)	PPV (3% prevalence)	PPV (6% prevalence)	PPV (10% prevalence)
Pre-BD FEV_1/FEV_6 ratio									
<0.4	6	0	131	407	1.5% (0.5%, 3.1%)	100% (97.2%, 100%)	-	-	-
<0.5	34	0	131	379	8.2% (5.8%, 11.3%)	100% (97.2%, 100%)	-	-	-
<0.6	89	0	131	324	21.6% (17.7%, 25.8%)	100% (97.2%, 100%)	-	-	-
<0.7	200	0	131	213	48.4% (43.5%, 53.4%)	100% (97.2%, 100%)	-	-	-
<0.71	210	1	130	203	50.9% (45.9%, 55.8%)	99.2% (95.8%, 100%)	0.67 (0.23, 0.94)	0.81 (0.38, 0.97)	0.88 (0.51, 0.98)
<0.72	226	1	130	187	54.7% (49.8%, 59.6%)	99.2% (95.8%, 100%)	0.69 (0.24, 0.94)	0.82 (0.39, 0.97)	0.89 (0.53, 0.98)
<0.73	242	1	130	171	58.6% (53.7%, 63.4%)	99.2% (95.8%, 100%)	0.70 (0.25, 0.94)	0.83 (0.41, 0.97)	0.90 (0.55, 0.98)
<0.74	255	1	130	158	61.7% (56.9%, 66.5%)	99.2% (95.8%, 100%)	0.71 (0.26, 0.95)	0.84 (0.42, 0.97)	0.90 (0.56, 0.98)
<0.75	268	2	129	145	64.9% (60.1%, 69.5%)	98.5% (94.6%, 99.8%)	0.57 (0.33, 0.78)	0.73 (0.51, 0.88)	0.83 (0.64, 0.93)
<0.76	285	4	127	128	69.0% (64.3%, 73.4%)	97.0% (92.4%, 99.2%)	0.41 (0.30, 0.53)	0.59 (0.47, 0.70)	0.72 (0.61, 0.80)
<0.77	297	5	126	116	71.9% (67.3%, 76.2%)	96.2% (91.3%, 98.7%)	0.37 (0.29, 0.46)	0.55 (0.45, 0.64)	0.68 (0.59, 0.75)
<0.78	304	6	125	109	73.6% (69.1%, 77.8%)	95.4% (90.3%, 98.3%)	0.33 (0.27, 0.40)	0.51 (0.43, 0.58)	0.64 (0.57, 0.71)
<0.79	320	8	123	93	77.5% (73.1%, 81.4%)	93.9% (88.3%, 97.3%)	0.28 (0.24, 0.33)	0.45 (0.39, 0.51)	0.59 (0.53, 0.64)
<0.8	335	8	123	78	81.1% (77.0%, 84.8%)	93.9% (88.3%, 97.3%)	0.29 (0.25, 0.34)	0.46 (0.40, 0.52)	0.60 (0.54, 0.65)
<0.9	408	107	24	5	98.8% (97.2%, 99.6%)	18.3% (12. 1%, 26.0%)	0.04 (0.04, 0.04)	0.07 (0.07, 0.07)	0.12 (0.12, 0.12)

TP = true positives, FP = false positives, TN = true negatives, FN = false negatives, sens = sensitivity, spec = specificity, PPV = positive predictive value

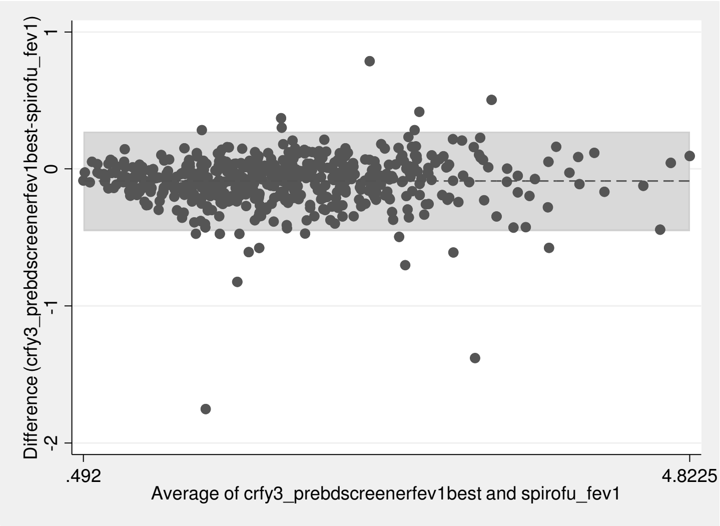

Supplementary figure 1: Bland Altman plot of FEV_1 agreement between pre-BD lung monitor and post-BD confirmatory spirometry

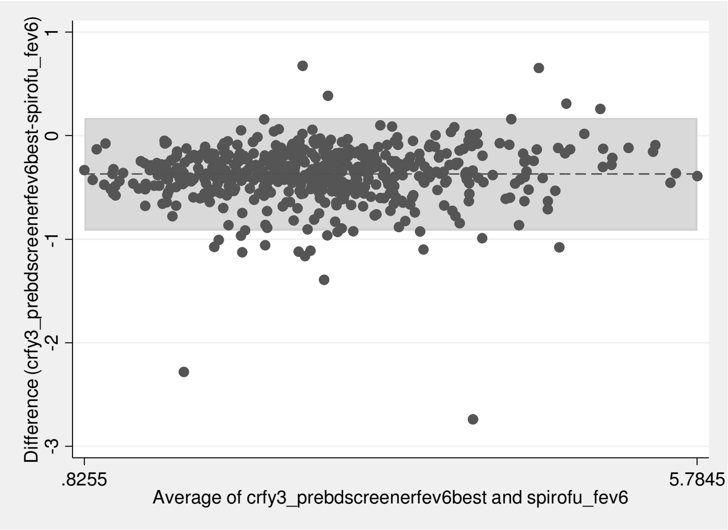

Supplementary figure 2: Bland Altman plot of FEV_6 agreement between pre-BD lung monitor and post-BD confirmatory spirometry

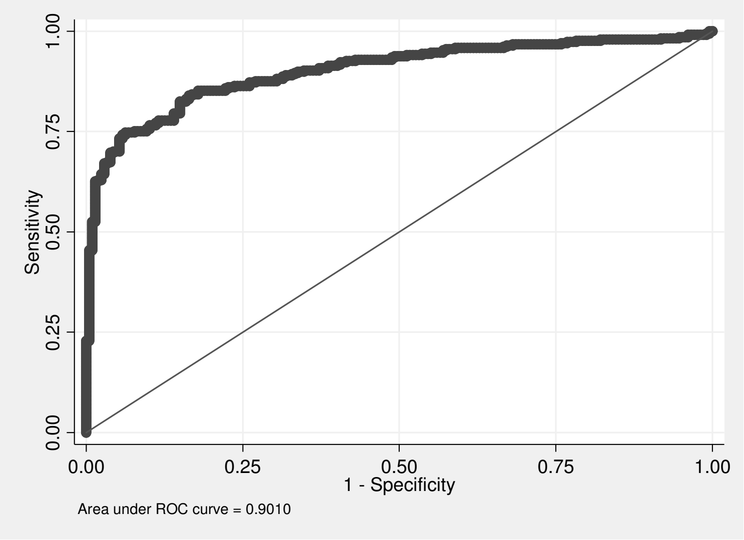

Supplementary figure 3: ROC curve for COPD diagnosis using pre-BD lung monitor (FEV_1/FEV_6 <LLN) against post-BD confirmatory spirometry (FEV_1/FVC <LLN)

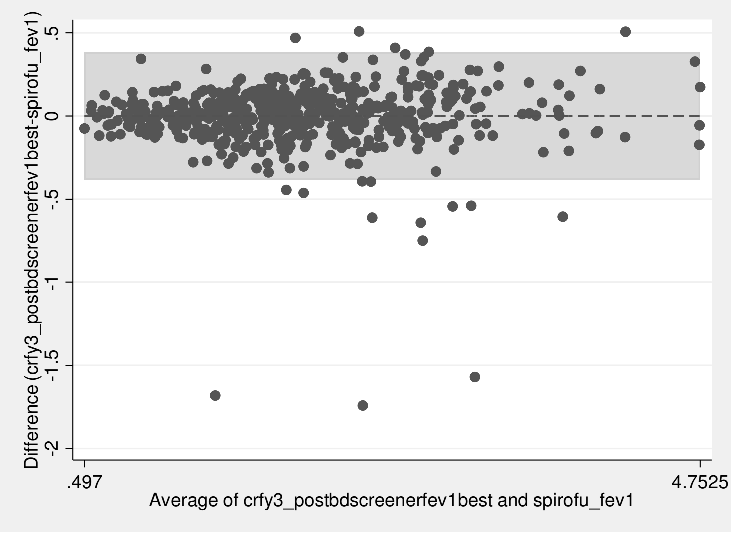

Supplementary figure 4: Bland Altman plot of post-BD FEV_1 agreement between lung monitor and confirmatory spirometry

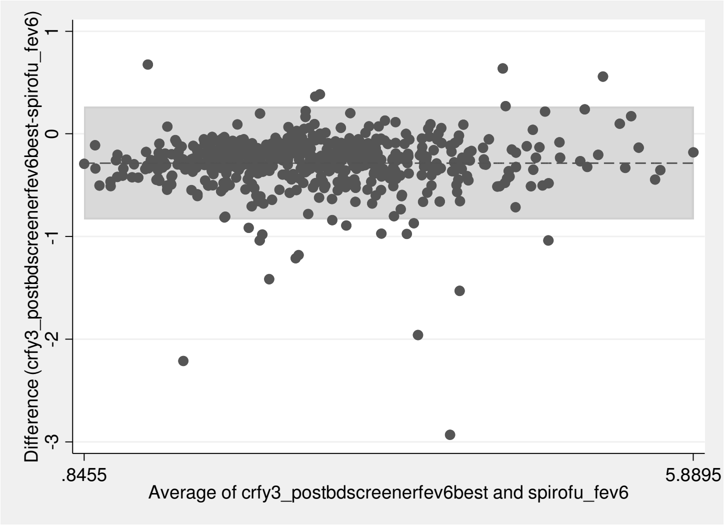

Supplementary figure 5: Bland Altman plot of post-BD FEV_6 agreement between lung monitor and confirmatory spirometry

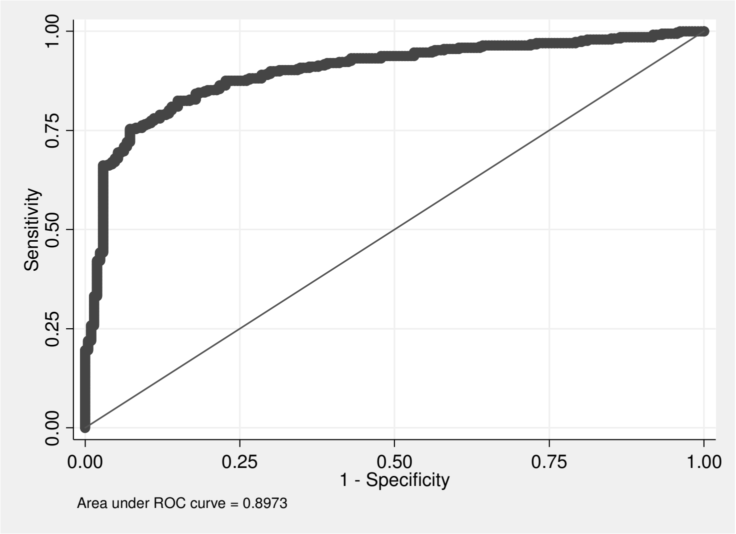

Supplementary figure 6: ROC curve for COPD diagnosis using post-BD lung monitor (FEV_1/FEV_6 <LLN) against post-BD confirmatory spirometry (FEV_1/FVC <LLN)

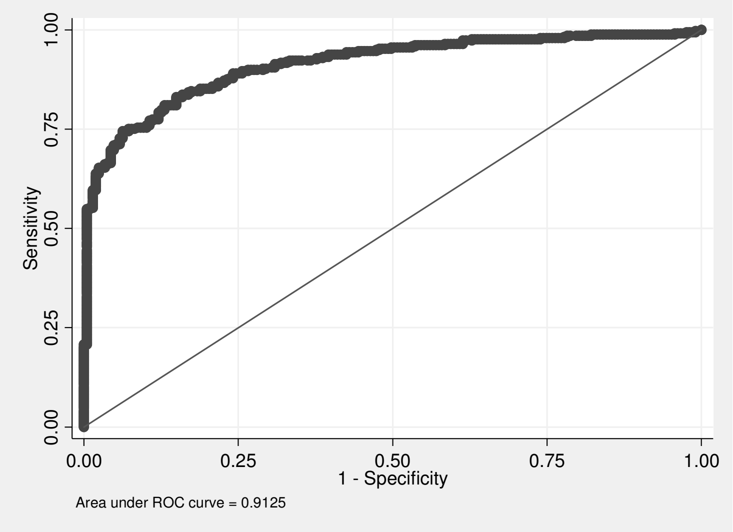

Supplementary figure 7: ROC curve for COPD diagnosis using pre-BD lung monitor (FEV_1/FEV_6 <0.7) against post-BD confirmatory spirometry (FEV_1/FVC <LLN)
